# Evaluation of the efficiency and effectiveness of independent dose calculation followed by machine log file analysis against conventional measurement based IMRT QA

**DOI:** 10.1120/jacmp.v13i5.3837

**Published:** 2012-09-06

**Authors:** Baozhou Sun, Dharanipathy Rangaraj, Sunita Boddu, Murty Goddu, Deshan Yang, Geethpriya Palaniswaamy, Sridhar Yaddanapudi, Omar Wooten, Sasa Mutic

**Affiliations:** ^1^ Department of Radiation Oncology Washington University School of Medicine St. Louis MO; ^2^ Department of Radiation Oncology Scott & White Healthcare System Temple TX; ^3^ Department of Radiation Oncology University of California Davis Sacramento CA USA

**Keywords:** quality assurance, IMRT, dose calculations, machine log file

## Abstract

Experimental methods are commonly used for patient‐specific IMRT delivery verification. There are a variety of IMRT QA techniques which have been proposed and clinically used with a common understanding that not one single method can detect all possible errors. The aim of this work was to compare the efficiency and effectiveness of independent dose calculation followed by machine log file analysis to conventional measurement‐based methods in detecting errors in IMRT delivery. Sixteen IMRT treatment plans (5 head‐and‐neck, 3 rectum, 3 breast, and 5 prostate plans) created with a commercial treatment planning system (TPS) were recalculated on a QA phantom. All treatment plans underwent ion chamber (IC) and 2D diode array measurements. The same set of plans was also recomputed with another commercial treatment planning system and the two sets of calculations were compared. The deviations between dosimetric measurements and independent dose calculation were evaluated. The comparisons included evaluations of DVHs and point doses calculated by the two TPS systems. Machine log files were captured during pretreatment composite point dose measurements and analyzed to verify data transfer and performance of the delivery machine. Average deviation between IC measurements and point dose calculations with the two TPSs for head‐and‐neck plans were 1.2±1.3% and 1.4±1.6%, respectively. For 2D diode array measurements, the mean gamma value with 3% dose difference and 3 mm distance‐to‐agreement was within 1.5% for 13 of 16 plans. The mean 3D dose differences calculated from two TPSs were within 3% for head‐and‐neck cases and within 2% for other plans. The machine log file analysis showed that the gantry angle, jaw position, collimator angle, and MUs were consistent as planned, and maximal MLC position error was less than 0.5 mm. The independent dose calculation followed by the machine log analysis takes an average 47±6 minutes, while the experimental approach (using IC and 2D diode array measurements) takes an average about 2 hours in our clinic. Independent dose calculation followed by machine log file analysis can be a reliable tool to verify IMRT treatments. Additionally, independent dose calculations have the potential to identify several problems (heterogeneity calculations, data corruptions, system failures) with the primary TPS, which generally are not identifiable with a measurement‐based approach. Additionally, machine log file analysis can identify many problems (gantry, collimator, jaw setting) which also may not be detected with a measurement‐based approach. Machine log file analysis could also detect performance problems for individual MLC leaves which could be masked in the analysis of a measured fluence.

PACS numbers: 87.53.Bn, 87.55.Qr, 87.55.km, 87.57.Uq

## I. INTRODUCTION

Since the introduction of intensity‐modulated radiotherapy (IMRT), the physical measurements and patient‐specific QA procedures to validate each IMRT plan before treatment have been considered an integral component of this delivery technique.^1^, ^2^ The comprehensive QA is essential for IMRT due to the complex nature of treatment planning and multitude of interfaces between the treatment planning system (TPS) and treatment delivery. Recommendations and guidelines for the appropriate implementation of IMRT and support of an adequate QA program to safely delivery IMRT treatments were provided in recent publications and reports.^2^, ^4^ Currently, experimental methods are predominantly used for patient‐specific IMRT delivery verifications. Typical measurement‐based procedures for pretreatment dosimetric verification include point dose measurements using ion chambers (IC) for a delivery including all treatment fields at the planned gantry angle, and 2D dosimetry measurement using radiographic films or 2D diode or ion chamber arrays at a vertical gantry angle for individual IMRT fields.^5^, ^9^

Traditional measurement‐based QA verification techniques may not be sensitive enough to detect many types of failures (such as plan transfer errors, beam delivery error, dose calculations errors) in the IMRT process.^10^, ^11^ Traditional IMRT QA processes rely on dose verification measurements in water equivalent plastic phantoms, which do not represent patient geometry or tissue heterogeneities. This oversimplification may not be able to identify calculation errors in some treatment sites. Additionally, creation of QA plans in the primary TPS requires recalculation of dose on the water equivalent phantom or 2D measurement array. This recalculation breaks the connection between the patient treatment plan and the QA plan, and any potential errors which were present in the calculation of the patient treatment plan may not be propagated to the phantom QA plan due to resetting of the calculation. Furthermore, experimental methods are time‐consuming and labor‐intensive, and require access to the treatment machine. There is a growing interest in using independent dose calculation^12^, ^15^ and machine log files^16^, ^18^ for QA of IMRT delivery. Dietmar et al.^(13)^ have proposed a semi‐analytical fluence based dose calculation for patient‐specific monitor unit (MU) verification. Monte‐Carlo–based independent dose calculations have been suggested for routine IMRT verification^(14)^ and even to replace the dosimetric verification in phantom.^(15)^ In addition to research and clinical interest, the commercial interest in independent dose calculation for IMRT is growing and tools have become commercially available.^19^, ^20^ Recently there is still a debate to evaluate the necessity and effectiveness of dosimetric validation of each individual IMRT treatment plan with dosimetric plan before delivery.^(12)^ The machine log file analysis has been proposed as an alternative for IMRT QA by several groups.^(16)^ Log files have been used to study step‐and‐shoot and dynamic MLC deliveries.^17^, ^18^ A commercial software that automatically verifies delivery accuracy for patient treatment using the machine log files has become available. In our department, log file analysis has been a routine component of patient‐specific IMRT QA procedure for several years.^(21)^

It has been shown that no single QA technique can mitigate all the errors that can happen in the IMRT process.^22^, ^23^ Table 1 shows a qualitative analysis of relative effectiveness of different IMRT verification for catching errors that can happen in IMRT delivery. It is critical to evaluate the efficiency and effectiveness of traditional QA approaches, and explore the possibility of augmenting or replacing current QA techniques with more effective, systematic, reliable, and efficient methods. In this work, we have evaluated the efficiency and effectiveness of independent dose calculation in combination with machine log files analysis as an approach to IMRT QA. We accomplish this by:

**Table 1:** A qualitative analysis of effectiveness of QA techniques to catch some potential discrepancy or error that could happen in an IMRT treatment. Note: only a few are mentioned here and only pretreatment QA techniques are analyzed.

*Data Transfer, Delivery Error, Planning Error Type*	*Point Dose Measurement* ^*a*^	*Field‐by‐Field Planar Dose QA* ^*b*^	*Composite Planar Dose QA* ^*c*^	*DynaLog QA* ^*d*^	*Independent Dose Calculation QA* ^*e*^
**Beam Parameters Discrepancy**					
**During Data Transfer or Machine Delivery**					
Gantry Angle	3	5	4	1	5
Collimator Jaw Setting	3	3	3	1	5
Collimator Angle	3	3	3	1	5
MLC Positioning Error	4	3	3	1	5
MUs	1	1	3	5	5
Couch Angle Error	2	5	2	5	5
**Machine Issues/Data Transfer Issues**					
Dosimetry Characteristic –					
Energy Change, Symmetry and	4	4	4	5	5
Flatness Off					
Absolute Dose Output Calibration	5	5	5	5	5
Relative Dose Output – Small Field Output Off	1	1	1	5	5
One Segment Dropped Out or Not Transferred Properly	4	3	4	1	5
One Field Not Transferred Correctly	4	2	3	1	5
Demanding MLC Sequence or MLC Positioning Issues – Beam Hold Off	4	4	4	1	5
**TPS Beam Modeling Issues**					
Small Field Out Prediction Issue	2	2	2	5	1
Heterogeneity Correction Issues	5	5	5	5	1
Wrong CT to ED	5	5	5	5	2
DVH Calculation Discrepancy	5	5	5	5	1
***In vivo* Changes**					
Beam Data Modification After					
Pretreatment QA and Other Machine Issues During Each Fraction	5	5	5	5	5
**IGRT Issues**					
Anatomy Changes, localization Issues, Setup Issues	5	5	5	5	5
**Treatment Planning**					
Isocenter Placement, Prescription, Wrong CT Voxel Size, Plan Quality	5	5	5	5	5

Note: 1 is most effective, 4 is least effective, and 5 is not possible to find from QA test results.

^a^ Point dose measurement refers to ion chamber measurement with one or two points in a composite fashion (i.e., all beam delivered to a water equivalent phantom as it would be delivered to the patient).

^b^ Field‐by‐field planar dose measurement: all beams delivered from AP direction with gantry and could reset to default position.

^c^ Composite planar dose QA refers to measuring a plane using a 2D detector embedded in a phantom and the QA is performed with actual beam parameters as it will be delivered to the patients.

^d^ DynaLog QA: analysis of machine log file collected by delivering the actual plan to air or during composite point or planar dose measurement, as explained in a) and c).

^e^ Independent dose calculation is verifying the dose distribution of the planning system by recalculating in an independent dose calculation by exporting DICOM RT files (Plan, Dose, Images, Structure set) and any POIs.

a) Comparing the efficiency and accuracy of dose calculation and machine log file analysis QA paradigm against current measurement based technique.
b) Evaluating the potential of two paradigms for error detection effectiveness and reliability.

To do this, we chose to use another commercial treatment planning system to verify the IMRT dose distribution computed by the primary treatment planning system. Independent dose calculation does not verify the MLC performance during IMRT delivery, although it may reduce or reveal errors/inconsistencies in the treatment planning system and process. We propose to perform the machine log file analysis to supplement the calculation‐based QA to validate data transfer and delivery performance of MLCs.

## II. MATERIALS AND METHODS

### A. Treatment plans

In the present study, 16 IMRT plans have been evaluated by experimental methods and by an independent dose calculation method followed by machine log file analysis. Treatment plans with fixed beams and static multileaf collimator (SMLC) IMRT treatment plans were chosen for this study. Treatment sites and number of plans included in the study were: 5 head‐and‐neck plans, 3 rectum plans, 3 breast plans all using 6 MV, and 5 prostate plans using 18 MV. The average number of beams used in this study for head‐and‐neck, rectum, breast, and prostate were 9, 9, 10, and 7, respectively. Treatment plans were created using Pinnacle 9.0 TPS (Philips Medical Systems, Fitchburg, WI). The number of segments used per beam varied between 4–12, while the total number of segments varied between 40 and 78 per treatment plan. All plans were optimized using direct machine parameter optimization (DMPO).^(24)^

### B. Verification procedures

#### B.1 Traditional measurement‐based IMRT QA

**Figure 1:**
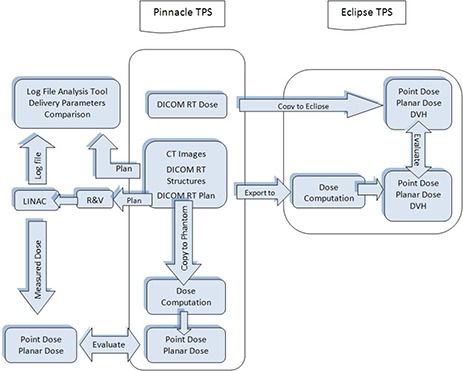
Process flow for independent dose calculations with machine log file analysis and measured‐based QA in our clinic.

Figure 1 shows the entire verification process presented in this paper (steps grouped inside the black box). After an IMRT treatment plan was approved for treatment, it was recalculated using the same beam orientations and MUs, but replacing the patient CT dataset with a water equivalent phantom $\left(14\times 14\times 15{\;\text{cm}}^{3}\right)$. The phantom has inserts to accommodate two small‐volume ionization chambers (IC) anywhere within the box at a 0.5 cm resolution.^(6)^ Two IC point measurements were performed for each approved plan. Additionally, planar dosimetry QA for each field of each plan was performed using a 2D diode array (MapCHECK, Sun Nuclear Corporation, Melbourne, FL) mounted using a custom‐built water equivalent plastic phantom. The MapCHECK device consists of 445 N‐type diodes that are in 22 by $22\;{\text{cm}}^{2}$ 2D array with variable spacing of 7 and 14 mm between diodes. Calculated planned fluences were copied onto the verification phantom in such a way that the high‐dose region is located in the central area where there is high detector density of the MapCHECK device. The verification measurements were performed using a static gantry that is perpendicular to the measurement plane. The measured fluence maps of the individual beams were compared to the fluence maps computed by the TPS. The goal was that IC measurements should verify the absolute dosimetry, while the planar dosimetry measurements validate the relative dose distributions of the individual beams.

#### B.2 Calculation‐based verification method

The proposed calculation‐based verification method consists of independent dose calculation and machine log file analysis. The process for independent calculation‐based QA is also shown in the flowchart of Fig. 1. All treatment plans were generated on the Pinnacle treatment planning system. DICOM RT files which include RT dose, RT plan, RT structures, and CT images were exported to another commissioned and clinically used commercial treatment planning system (Eclipse V 8.2.0, Varian Medical Systems, Palo Alto CA). The dose distributions were recalculated using Eclipses' analytical anisotropic algorithm (AAA).^(25)^ The point doses and DVHs were exported in DICOM format and analyzed in Eclipse TPS. The planar dose files from both treatment planning systems were exported into I'MRT MatriXX (IBA dosimetry, Bartlett, TN) for qualitative and quantitative gamma analysis. The use of independent dose calculation is an alternative method to evaluate the accuracy of the treatment planning system's dose calculations, including heterogeneity corrections.

The Pinnacle plan was calculated using Collapsed Cone Algorithm with dose grid size of 3 mm×3 mm×3 mm while, for Eclipse treatment planning system, Anisotropic Analytical Algorithm was used with a dose grid size of 2.5 mm×2.5 mm×3 mm. Both the systems have implemented convolution superposition algorithms in their own way. The dose‐volume histograms (DVH) were computed using Eclipse TPS for both Pinnacle and Eclipse dose grids. The DVHs and the point doses of two plans were then evaluated for PTV and critical structures.

The machine log files were captured during pretreatment ion chamber QA measurements. Varian linear accelerators (linac) write the actual machine parameters every 50 ms, and store them in a file (DynaLog) on the linac control console and MLC workstation. We used log files from Varian Trilogy and iX machines which record machine parameters every 50 ms, while the Varian TrueBeam machine, which records machine parameters every 10–20 ms, was not considered for this study. The DynaLog files include beam on/off status, gantry angle, collimator angle, jaw positions, and MLC leaf positions for all control points and delivered beam MU, etc. These log files are accessible after the delivery of each fraction of the treatment.

After an IMRT treatment plan is approved, it is exported to the R&V system and delivered on the linac machine for point dose (IC) QA measurements, and the machine log files stored during the delivery were transferred to and stored in a folder on a network drive. In this study, we capture the DynaLog files that were recorded during pretreatment point dose measurement. In a parallel path, the treatment plan was exported as a DICOM file containing the planned values of the machine parameters to the same network drive folder. Semi‐automatic machine log file analysis is performed using in‐house–developed MATLAB (MathWorks Inc., Natick, MA) software which compares the patient identification information (name, ID number, etc.), log file integrity, and the delivered machine parameters for each beam with the respective planned values. Beam parameters include gantry angle, collimator angle, number of segments, and MLC leaf positions for all control points. In addition, based on the beam on/off status and MLC leaf positions retrieved from the machine log files, the software computes the equivalent fluence map (in MUs) at the isocenter for each delivered beam, compares it against the planned beam fluence map reconstructed using the same algorithm, and calculates pixel‐by‐pixel fluence difference between the two maps. Finally, the software generates a summary report of all the beam parameters, and three fluence maps for each beam. The report is color‐coded and includes warning messages for parameters that are outside specified tolerances.

## III. RESULTS

### A. Evaluation of measurement based IC and planar dosimetry IMRT QA

In the past three years, we have performed ~4000 IMRT QAs with ion chambers, field‐by‐field planar dosimetry and composite planar dosimetry. We have not found any errors that would result in replanning of the patient treatment or modification of treatment delivery parameters. We have treated variety of different sites with SMLC and DMLC technique. However, we have repeated ~15% of the QA measurements because of incorrectly generated/delivered QA plan, equipment failures, selected points being in high dose gradient regions, or wrong documentation of QA plan (shifts or point coordinates), resulting in additional time and resources. While this is true, it doesn't mean that there were no discrepancies between planned and delivered treatments. We have found instances where there were discrepancies in the R&V system that were caused by data entry or other human errors, and for which the pretreatment QA methods were insensitive and therefore did not catch, but that were discovered during initial and weekly chart checks.

### B. Validation of independent dose calculation and machine log QA paradigm with measurements

The IMRT phantom plans were verified with ICs and MapCHECK. For composite IMRT plan deliveries, average deviation between IC measurements and point dose calculation with Pinnacle and Eclipse for head‐and‐neck plans for all the selected patients were 1.2±1.3% and 1.4±1.6%, respectively (Table 2). MapCHECK measurement and dose distribution computed from Pinnacle were compared using the gamma evaluation method with 3% dose difference and 3 mm distance‐to‐agreement as acceptance criteria. Dose distributions obtained from Pinnacle were used as reference. Table 2 also lists the average gamma passing rate for the various treatment sites.

**Table 2:** Ion chamber measures to validate independent dose calculation technique.

	*Eclipse Calculated Dose / Pinnacle Calculated Dose (Avg ± STD)*		*Measured / Pinnacle Calculated Dose (Avg ± STD)*		*MapCHECK Gamma Passing Rate (Avg ± STD) %*
*Treatment Site*	*IC1*	*IC2*	*IC1*	*IC2*	
Head‐and‐ Neck	0.972±0.005	0.980±0.009	1.022±0.008	1.013±0.010	96.3±1.62
Breast	0.990±0.004	0.996±0.01	1.010±0.006	1.009±0.020	94.8±1.13
Prostate	0.995±0.01	1.01±0.004	0.994±0.009	0.997±0.012	98.2±0.58
Rectum	0.975±0.008	1.005±0.01	1.017±0.016	1.017±0.025	94.9±1.68

**Figure 2:**
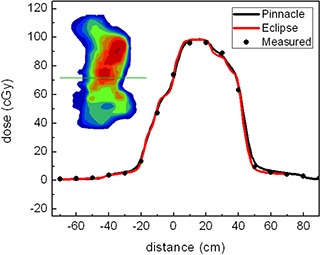
Line dose profiles for an H&N IMRT plan verified by MapCHECK for Pinnacle and Eclipse calculations.

**Figure 3:**
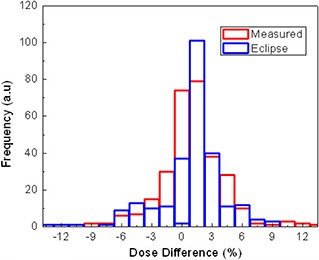
Dose difference derived from the difference between measurements vs. Pinnacle and Eclipse calculations vs. Pinnacle.

Figure 2 represents typical line dose profiles of the calculations and measurements for a single head‐and‐neck (H&N) treatment field. The insert shows the dose distribution and the position of the line profile. Both the calculated dose distribution using Eclipse and measured using MapCHECK match quite well with the calculated from Pinnacle. Figure 3 shows the histograms of the deviations of the MapCHECK measurements and Eclipse calculations compared to Pinnacle calculated IMRT plan on a pixel‐by‐pixel basis for the H&N patient presented in Fig. 2. The results demonstrate a little broadening of measurement data and not in the eclipse calculations. The broadening of the MapCHECK measurements may be from the small errors in positioning the phantom and the resolution of the diode array.

**Figure 4:**
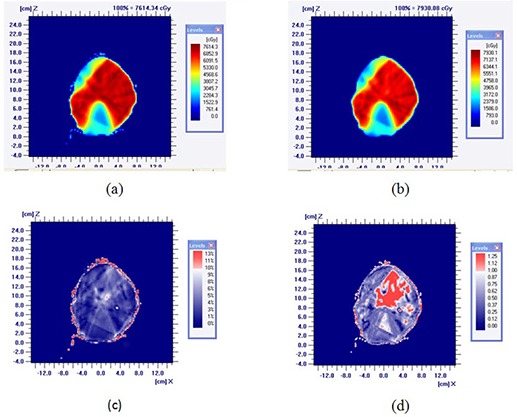
Dose map of a representative H&N case obtained with Pinnacle and Eclipse: (a) from Pinnacle; (b) from Eclipse; (c) difference; (d) 2D gamma.

The dose calculations using two different treatment planning systems were also compared based on the treatment site. Figure 4 shows the dose map of a representative head‐and‐neck case obtained with Pinnacle and Eclipse. (Figure 4c) and (d) shows the difference in dose and in gamma function analysis. The 2D gamma evaluation quantity was calculated using 3% dose and 3 mm spatial acceptance criteria. Table 3 shows the DVH indices for head‐and‐neck and rectum corresponding to Pinnacle and Eclipse. The mean dose differences are within 2% for both cases. In the same way, Table 4 lists the DVH indices for breast and prostate cases. The independent dose calculation deviates from Pinnacle by less than 1.8% for both PTV and critical structures.

**Table 3:** DVH indices for head‐and‐neck and rectum cases.

		*Head‐and‐Neck*		*Rectum*	
*PTV & Critical Structures*	*Dose Indices*	*Pinnacle (Avg.±SD)*	*Eclipse (Avg.±SD)*	*Pinnacle (Avg.±SD)*	*Eclipse (Avg.±SD)*
	V 95%	98.7±1.3	96.9±1.7	96.9±5.4	95.8±7.3
	V 105%	34.3±33.2	23.4±25.3	16.7±25.3	16.1±26.5
	V 107%	14.3±17.7	8.2±10.0	5.3±9.0	5.7±9.8
PTV1	Min. dose %	96.9±2.0	95.0±1.9	96.3±4.9	95.9±5.1
	D5%‐95%	8.9±2.8	10.0±2.9	7.4±3.2	7.5±3.1
	D3%‐93%	8.4±2.5	9.8±2.7	7.2±3.1	7.4±3.0
	Mean dose (cGy)	7236.3±140.3	7143.0±172.3	4281.1±1543.0	4270.7±1550.6
	V 95%	99.6±0.0	99.1±0.3	98.8±1.2	98.5±1.5
	V 105%	69.3±11.6	58.1±7.3	72.8±9.4	71.0±11.2
	V 107%	51.9±12.6	41.2±6.7	62.1±8.5	60.3±10.1
PTV2	Min. dose	97.0±0.2	95.3±0.7	95.8±4.2	95.3±4.8
	D5%‐95%	20.4±5.0	20.7±4.8	21.7±9.0	21.6±8.7
	D3%‐93%	22.0±5.4	22.4±4.9	21.4±9.1	21.3±8.7
	Mean dose (cGy)	5937.7±109.60	5862.2±128.3	4035.1±1566.2	4025.2±1570.7
Lt. Parotids	V26Gy	62.0±32.1	59.9±30.7	–	–
	Mean dose (cGy)	3586.6±1801.1	3494.6±1736.3	–	–
Rt. Parotids	V26Gy	53.7±40.4	52.3±40.7	–	–
	Mean dose (cGy)	3122.4±2020.3	3068.4±2016.6	–	–
Spinal Cord	Max. dose	3762.1±260.6	3599.2±257.2	–	–
Small Bowel	V40Gy	–	–	582.0±988.4	595.7±1012.2
	Mean dose (cGy)	–	–	1780.2±1102.2	1772.2±1097.8

**Table 4:** DVH indices for breast and prostate cases.

		*Breast*		*Prostate*	
*PTV & Critical Structures*	*Dose Indicies*	*Pinnacle (Avg.±SD)*	*Eclipse (Avg.±SD)*	*Pinnacle (Avg.±SD)*	*Eclipse (Avg.±SD)*
	V 95%	89.9±12.5	77.9±23.5	100.0±0.1	100.0±0.0
	V 105%	3.6±5.1	1.5±2.1	32.1±31.4	47.7±37.9
	V 107%	0.6±0.8	0.2±0.2	1.9±2.6	12.4±17.0
PTV	Min. dose	95.4±0.9	94.0±1.4	100.7±0.6	100.8±1.3
	D5%‐95%	8.1±0.1	8.8±0.3	3.1±1.4	3.6±1.7
	D3%‐93%	8.1±0.1	8.7±0.2	3.1±1.3	3.5±1.6
	Mean dose	4988.5±148.4	4923.2±147.6	6779.6±34.8	6828.6±50.1
Ipsilateral Lung	V20	26.3±3.8	26.0±3.9	–	–
	Mean dose (cGy)	1363.4±63.1	1375.4±66.5	–	–
Heart	V20	6.7±9.5	6.8±9.7	–	–
	Mean dose (cGy)	982.7±506.2	965.0±524.0	–	–
Spinal Cord	Max. dose (cGy)	1241.7±354.1	1267.3±323.1	–	–
	V65	–	–	10.3±1.4	10.5±1.3
Rectum	V40	–	–	27.1±1.8	27.3±2.0
	Mean dose (cGy)	–	–	2721.6±171.6	2743.2±152.0
	V65	–	–	15.6±5.6	16.0±5.6
Bladder	V40	–	–	25.9±6.8	26.6±6.9
	Mean dose (cGy)	–	–	2408.5±534.0	2461.7±529.9

**Figure 5:**
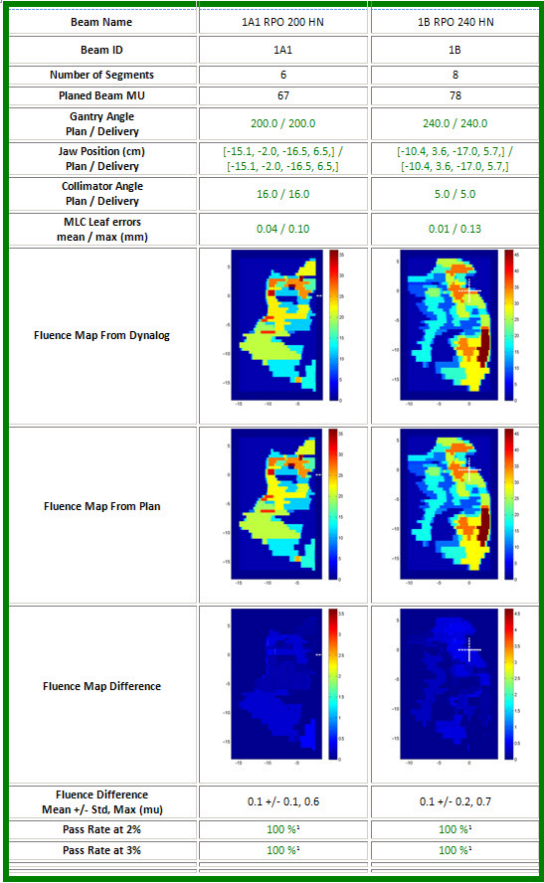
Machine log analysis. Gantry, jaw, and collimator angle, MLC positions, MU, and fluence map are compared.

It has been pointed out that independent dose calculations based on the exported DICOM file from Pinnacle to Eclipse do not check the potential error in the actual MLC performance of the treatment unit and accuracy of the delivery. The machine log files analysis for two beams delivered for a head‐and‐neck patient, shown in Fig. 5, reveals the fluence maps of planned and delivered (without considering the scattering) and their difference. This report also includes the status of several beam parameters including gantry angle, collimator angle, jaw positions, delivered MU, and MLC errors. If the actual parameters are within the set tolerance, the check results are displayed in green, otherwise as a “warning” in yellow. The tolerances for gantry angle, collimator, jaw position, and MLC leaf positions are 0.1°, 0.1°, 1 mm and 2 mm, respectively. We have found that the maximal MLC errors for the 16 patients were less than 0.5 mm. The passing rate at 2% (3%) means the percentage of the number of pixels with error less than the 2% (3%) of the maximal fluence (in MU) of the entire beam.

### C. Efficiency of independent dose calculation and machine log file analysis paradigm

Table 5 shows the comparison of the total time for experimental IMRT verification in solid phantom, based on IC and MapCHECK measurements, and calculation‐based approach. The independent dose calculation with the machine log analysis can be done during the day and only takes 32 minutes. This time comparison favors the independent dose calculation, as the experimental approach takes about 2 hours.

**Table 5:** Process flow timeline.

*Steps*	*Time Scale*	*Experimental‐based Approach*	*Computation‐based Method*
1	Making a verification plan for measurements (IC and 2D verification plans)	35 min	15(capturing DynaLog)
2	Exporting verification plan for delivery	3 min	‐
3	1D and 2D verification at LINAC	60 min	‐
4	Data analysis of the measurement data	15 min	‐
5	Export the plan from Pinnacle to Eclipse	‐	10 min
6	Independent dose calculation in Eclipse	‐	8 min
7	DVH comparison and analysis	‐	6 min
8	Machine log analysis	‐	3 min
9	Documentation	5 min	5 min
Total Time		118 min	47 min

## IV. DISCUSSION

There are many potential errors that can arise during the IMRT planning and delivery, such as dose calculation inaccuracies, plan transfer errors, beam delivery errors, patient setup errors, and target location uncertainties due to organ motion. A comprehensive quality assurance program should be established to efficiently check these potential errors and to ensure that dose distribution planned on an IMRT treatment planning system will be delivered accurately and safely. Current patient‐specific QA techniques are performed by irradiating a water equivalent phantom that contains film, IC arrays or equivalent dose measurement tools to verify that the dose delivered is the dose planned. These methods can detect large errors in beam delivery, but might not detect dose calculation errors. Furthermore, the verification measurements are based on the homogenous phantom, which does not take into account the heterogeneities in patient's anatomy. The measurement‐based QA methodology in water equivalent phantoms may not catch the errors associated with lack of electronic equilibrium caused by small segments in the presence of heterogeneities. The independent dose calculation can catch the serious errors in heterogeneity calculation or beam modeling.

It has been recognized that today's patient‐specific measurement‐based QA has serious limitations and it is also time‐consuming and labor‐intensive. The value of validating individual plans has been questioned, and there has been a debate whether validating treatment plan is worthwhile after the commission process has been completed.^12^, ^26^, ^27^, ^28^ With IMRT, proper commissioning will help avoid most systematic errors. Independent dose calculation can serve as an alternative verification method which can free up time for the physicist to evaluate the entire scope of an IMRT treatment. The other advantage of independent dose calculation method is that the linear accelerator time is not required. And the independent dose calculation and machine log file analysis can be done, on average, in 32 minutes, which is much more efficient than the measured‐based QA approach. The efficiency of the independent dose computation and log file analysis method can be improved by designing a fully automatic QA tool. A comprehensive commercial solution that would automate the whole process will make it even more efficient.

In this study, we have used the 2D gamma to evaluate the dose distribution accuracy between planning systems, but a full 3D gamma evaluation would be useful. 3D gamma can also aid us finding discrepancy in areas that are not contoured as structures where discrepancy cannot be figured out from DVH metrics. This 3D gamma tool would add additional robustness to the independent dose calculation‐based QA paradigm.

We used a commercial treatment planning system as an independent dose calculator instead of Monte Carlo method because Eclipse is a FDA‐approved commercial treatment planning system and the dose calculations on this system are reasonably fast. The use of Monte Carlo has been proposed by several authors,^29^, ^30^ but we avoided that for two reasons: i) we believe the goal of the independent dose calculator is to act as a QA tool looking for major errors instead of determining the accuracy of the commissioned treatment planning system algorithm and beam model; ii) from an efficiency aspect, the time required to compute independent dose calculations with Monte Carlo are prohibitively long.

It has been realized that the independent dose calculation alone is not sufficient for a comprehensive QA program because the data transfer from the TPS to the linear accelerators and the performance of the delivery unit are not checked. Therefore, a periodic QA testing of the machine delivery accuracy is required. Computer‐based machine log file analysis should catch any errors associated with plan transfers and the delivery problems. The machine log file analysis should authenticate the delivery quality. Also the machine log file QA is a number‐to‐number comparison of data transferred from treatment planning system to data delivered by the linear accelerator and is more sensitive to detecting discrepancies of the order or 1 mm and 1 degree compared to measurement‐based method. The sensitivity of IC and planar dose measurements is determined by volume of ion chambers and resolution of MapCHECK or MatriXX. In our clinics, the machine log file QA have detected data transfer errors, jaw position errors, and MLC errors which were not caught in IC and planar dose measurements.^(21)^ Most of these errors were related to the data transfer and human error, and the sensitivity and efficiency of the IC and planar dose measurements in mitigating errors were revealed.^(21)^ This work also leads us to believe that current QA methodology has short comings.

The workflow for Pinnacle patients using verification by independent dose calculation method is to export the clinically approved treatment plans to Eclipse planning system to perform independent dose calculations using the AAA algorithm. If the Eclipse dose‐volume indices for PTVs and OARs match the Pinnacle TPS within 3%, the patient should go for treatment without QA measurements, and one can perform machine log file analysis on the patient's first treatment fraction to validate the delivered treatment beam parameters against the planned. The benefit of doing machine log file analysis on first fraction is that there is no delay in process because of QA, and the treatment can resume immediately after the plan is ready. Also, any changes to the treatment record after pretreatment checks are made could be detected. The downside of this approach is that, if there are any errors in the beam parameters prepared for delivery, the error would be found only after delivering a dosimetrically different dose than the plan for the first fraction. Therefore, this methodology may not be suitable for single fraction treatments as well as hypofractionated ESRT treatments. For this case, a QA beam could be delivered and the machine log file analysis could be done before the patient treatment. Even if a pretreatment IC QA is performed, it would be a better practice to perform machine log QA analysis after first fraction to ensure nothing has changed after QA and initial approval. One could also evaluate a paradigm doing DynaLog QA, which is performed after every field to reduce the risk. Also DynaLog QA can be performed on pretreatment and also on the first fraction as an *in vivo* measurement. In our clinic, we are performing DynaLog QA for every field, for every fraction, and for every IMRT patient, to study the effectiveness of that paradigm,^(31)^ which will be reported in a separate manuscript. The method of choice should be based on effectiveness, efficiency, and safety of the delivery to the patients.

The paradigm shift from measurement‐based patient‐specific QA verification to a computation‐based methodology may have significant gain on the QA timeline and workflow, particularly towards implementation of adaptive radiation therapy. However, the transition should be gradual so as to provide enough confidence in IMRT verifications. At the start of clinical implementation of any new IMRT technique, it is strongly recommended to perform measurements of the 3D dose distribution delivered to a phantom and to compare with planned dose distributions. It should be kept in mind that the computation‐based methods do not exclude the measurement‐based methods for some cases. When the independent dose calculations and machine log files analysis yield an unacceptable result or when the independent dose calculations do not give satisfactory results, the experimental‐based measurement or other investigation should be systematically performed to track the errors. Also, at the time of upgrade or when any major component of treatment planning program is upgraded, it is advised to perform measurement‐based QA. Since measurement‐based methods are typically performed on homogeneous phantom, an independent dose calculation with heterogeneity correction should also be performed.

To implement the calculation‐based method as a QA approach using Eclipse, the independent dose calculation software has to be modeled accurately because the results of independent IMRT dose calculation are dependent on the leaf transmission, rounded leaf ends, and the tongue‐and‐groove effect. It should be noted that dose calculation discrepancy does exist due to differences between the TPSs treatment of heterogeneities (collapsed cone vs. AAA). The verification by independent dose calculation shows the difference in dose calculations between the Pinnacle and Eclipse treatment planning systems is within ±3% for head‐and‐neck, prostate, breast, and rectum cases. For sites in which tissues are nearly homogeneous (prostate), little heterogeneity error would be expected from independent of the dose calculation algorithm. For heterogeneous geometries (lung, head‐and‐neck), the heterogeneity errors would be expected to be greater. These were also seen in Tables 3 and 4 from our study. Approximations or inaccuracies in the conversion from MLC leaf sequences to fluence or intensity maps to be used by the dose calculation algorithm in Eclipse and Pinnacle treatment systems could also lead the dose differences between the two treatment planning systems. Such discrepancies should be expected and understood, while variance from such discrepancy should be an indicator of errors. Depending on the limitation and implementation of the algorithms that are used, there could be large discrepancies in certain situations (e.g., lung tumors with small PTVs) with AAA and collapsed cone algorithms.^(32)^ Thus this technique has limitations, and in such scenarios one must apply it with an understanding of the differences in algorithms.

The goal of the study is not to propose the use or purchase of the second commercial planning system. The goal is to study different QA paradigms in a quest to understand the effectiveness and efficiency. We had several choices for independent dose calculation software. We consciously decided to use a commercial, extensively tested independent dose calculation system as the secondary TPS. The reason for this was to choose an equally robust and verified dose calculator to avoid introducing other uncertainties into the paradigm. We thought the best choice would be a commissioned FDA‐approved TPS which has been routinely used in our clinic for a several years.

This is a process that is shown to be effective and it is important to have independent dose calculation software as good as a commercial TPS at a reasonable cost to provide safe and quality treatments.

## V. CONCLUSIONS

The present verification procedure with independent dose calculation followed by machine log file analysis is a reliable tool to verify the IMRT treatment. It allows the assessment of dose distribution in the patient anatomy, which cannot be obtained with conventional measurements using ICs or MapCHECK in a homogenous phantom. It can verify not only the calculation inaccuracies, but also verify the data transfer and evaluate the performance of the MLC delivery. Machine log file analysis is a much more sensitive tool of data transfer/entry discrepancy than measurement‐based techniques. This method offers significant advantage in reducing the time needed for the QA and it is less labor‐intensive. With the IMRT QA program becoming more mature, independent dose calculations and machine log analysis may be used to replace or compliment experimental‐based verification methods.
